# Supplementary material for: Loop-Mediated Isothermal Amplification (LAMP): Potential Point-of-Care Testing for Vulvovaginal Candidiasis
Source: J Fungi (Basel). 2023 Dec 2;9(12):1159. doi: 10.3390/jof9121159 (PMC10744362; doi:10.3390/jof9121159)
Supplement: Supplementary file 1 [file jof-09-01159-s001.zip › Supplement table.pdf]

Table S1 Primer of LAMP

Primer	Sequence	Target
C.alb-F3	GTTGGCAAAAATATCAATGGTAA	Candida albicans
C.alb-B3	GTAAACTCAGAAGCTGGAAC	
C.alb-FIP	AGCTTGGAAGGCATCAATAATATCTGTGTGTATCGATGTTCTTTTAGATTCTGGT	
C.alb-BIP	GACGGTCAAGGTCATACTTTCTAGTGTGTTGAAATCTTGGCGTTGTTGTC	
C.gla-F3	TGGTAGTGAGTGATACTCGTT	Candida glabrata
C.gla-B3	CTTAAAGACGTCTGTCTGCC	
C.gla-FIP	GCAGATTAATAGAGAAGCTTGCGCTGAGTTAACTTGAAATTGTAGGCC	
C.gla-BIP	GCGGCGGGGGTTAATACTGTCACAAAACACTCACTTATCCCT	
C.par-F3	GCGATAAGTAATATGAATTGCAGAT	Candida parapsilosis
C.par-B3	GAAGTTTTGGAGTTTGTACCA	
C.par-FIP	AGGCATGCCCTTTGGAATACCTGTGTGATTCGTGAATCATCGAATCTTTG	
C.par-BIP	GTCATTTCTCCCTCAAACCCTCGTGTGTGCATTAGTTTATACTCCGCCTTTC	
C.tro-F3	GTTTGAGCGTCATTTCTCC	Candida tropicalis
C.tro-B3	CTGTTGGTTTCTTTTCCTCC	
C.tro-FIP	GTCGCTTAAAATAAGTTTCCACGTTTGTGTGGGGTTTGGTGTTGAGCAATA	
C.tro-BIP	TTGCTAGTGGCCACCACAATTGTGTGGCTTAAGTTCAGCGGGTAG	

Table S2 Test results for LAMP and CHROMagar

Methods	ITS sequence				Total
	C.albicans	C.glabrata	C.tropicalis	Others	
**LAMP**					
C.albicans	63	0	0	0	63
C.glabrata	0	13	0	0	13
C.tropicalis	0	0	4	0	4
Others	0	0	0	0	0
**CHROMagar**					
C.albicans	65	2	0	0	67
C.glabrata	0	9	2	0	11
C.tropicalis	0	0	3	1	4
C. krusei	1	2	0	0	3
Others	2	0	0	1	3
