# Supplementary figures and images for: Loop-Mediated Isothermal Amplification (LAMP): Potential Point-of-Care Testing for Vulvovaginal Candidiasis
Source: J Fungi (Basel). 2023 Dec 2;9(12):1159. doi: 10.3390/jof9121159 (PMC10744362; doi:10.3390/jof9121159)

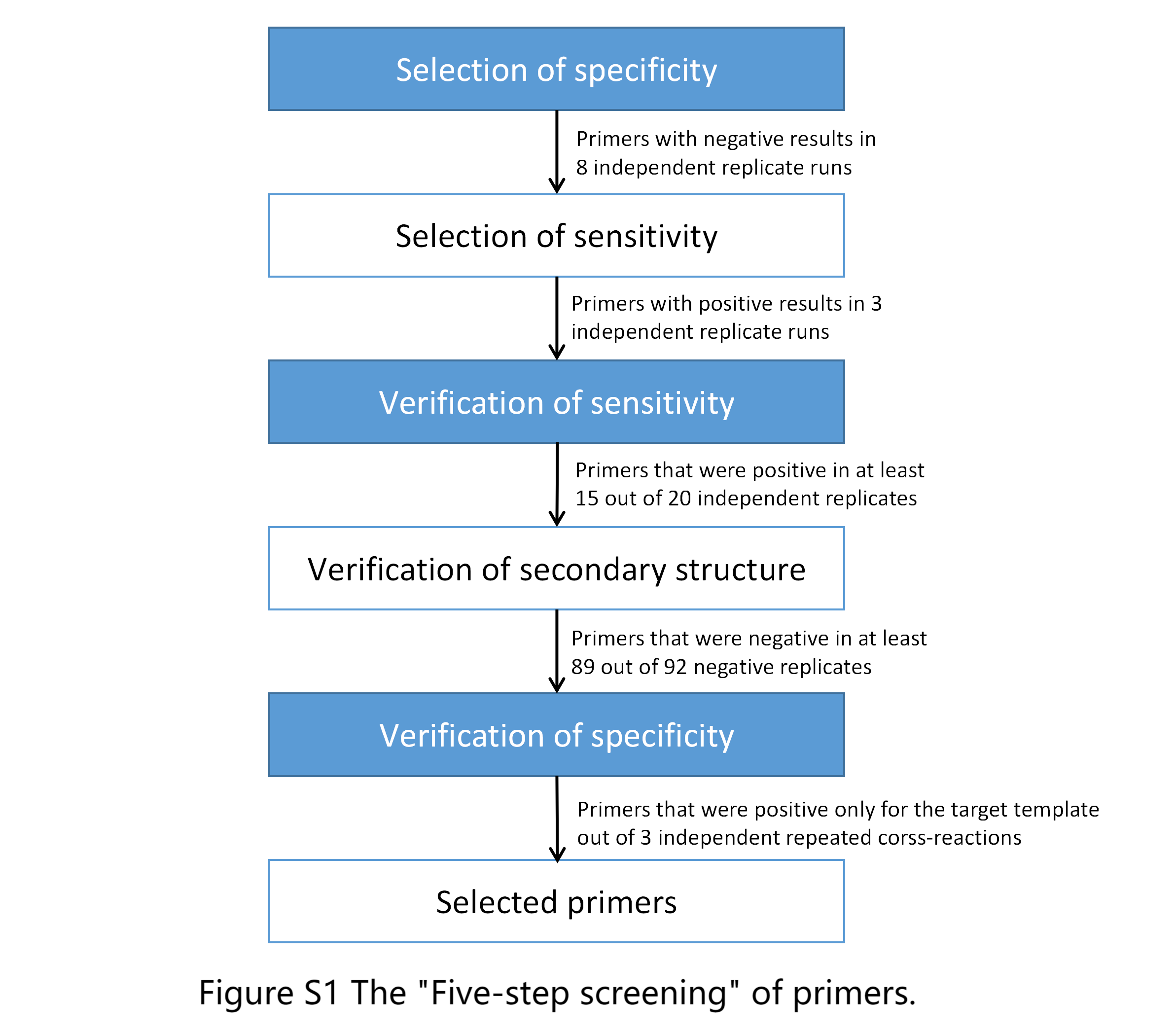

Supplement: Supplementary file 1 [file jof-09-01159-s001.zip › Figure S1.png]
